# Perception of Community and Hospital Personnel on Burn Treatment and Outcome

**DOI:** 10.31729/jnma.3889

**Published:** 2018-12-31

**Authors:** Jaswan Sakya, Sujit Kumar Sah, Khakindra Bahadur Bhandari, Laxmi Raj Pathak, Santosh Bikram Bhandari, Sudha Ghimire, Bhimsen Devkota, Jurgen Hussmann

**Affiliations:** 1 Sushma Koirala Memorial Hospital, Sankhu, Kathmandu, Nepal; 2 Tribhuwan University, Kritipur, Nepal; 3 Plastic and Reconstructive Surgery, Interplast Germany

**Keywords:** *burn injuries*, *hospitals*, *Nepal*, *outcomes*, *qualitative*

## Abstract

**Introduction:**

Globally, eleven million people sustain burn injuries every year enough to require medical attention. WHO has estimated Disability associated limited years of 84,000 per year just due to deformities and 2100 people die every year due to burn injuries in Nepal. The overall objective of the study is to explore the effectiveness of burn injuries treatment and management approach of hospitals.

**Methods:**

This qualitative study approached to 40 Health Personnel for Key Informants Interviews and 18 Focus Group Discussions with community people at the ten referral hospitals of eight district from May–June 2016. Qualitative data were analyzed using ATLAS.ti software.

**Results:**

Female burn victims are brought late to the hospital compared to male patients and false reporting about incident is usually done by her attendants. More than three-fourth (80%) of the hospitals and about one-third male and female from FGD reported that the community people seek home remedy first rather than medical treatment. Majority of the medical doctors and nursing chiefs reported that first degree cases accounts for 50% of the total burn cases with a success rate of 80%. Medical and Nursing staff reported that deformities like hypertrophic scar, keloids, joint stiffness and compartment syndrome are mostly observed during the treatment. Hypothermia and sepsis were the major causes of death in most of the burn patients.

**Conclusions:**

Usually, people who engaged in house and agriculture works, have visited public health posts/hospitals more frequently due to financial constraints and transportation issues where quality of burn care services are unavailable.

## INTRODUCTION

Globally, eleven million people sustain burn injuries every year enough to require medical attention.^1^ According to WHO, an estimated 180,000 deaths are caused annually due to burn injuries globally of which more than 50% of deaths occur South East Asia Region alone.^2,3^ Burns are the second most common injury in rural Nepal, accounting for 5% of disabilities. In Nepal, 55,902 sustain moderate to severe and 2100 people die every year due to burn injuries.^4^

Current medical facilities which offer treatment/ services related to deformities are rare and usually inaccessible to and unaffordable for the majority of people needing such kind of services. Furthermore, status of burn is unknown that directly have implication over planning and budget allocation for the prevention and management of burns.

The overall objective of the study is to explore the effectiveness of burn injuries treatment and management approach of hospitals in perspective of community people and health personnel.

## METHODS

The study was a qualitative cross-sectional study, based on primary information. This qualitative study approached to Key Informants interview (KII) and Focus Group Discussion (FGD) with Health Service Providers (HSPs) and community people respectively in the ten referral hospitals and their catchment areas of eight districts across the country from May to June 2016.

Ten referral hospitals for KIIs with HSPs were: 1. Koshi Zonal Hospital, Biratnagar, Morang, 2. BP Koirala Institute of Health Sciences, Dharan, Sunsari, 3. Narayani Sub-Regional Hospital, Birgunj, Parsa, 4. Bharatpur Hospital, Chitwan, 5. Tribhuwan University Teaching Hospital (TUTH), Maharajgunj, Kathmandu, 6. Bir Hospital, Kathmandu, 7. Sushma Koirala Memorial Hospital, Sankhu, Kathmandu, 8. Gandaki Sub-Regional Hospital, Pokhara, Kaski, 9. Bheri Zonal Hospital, Nepalgunj, Banke and 10. Seti Zonal Hospital, Dhangadhi, Kailali. Similarly, community people (both male and female) from the selected 10 hospitals' catchment areas were included for FGDs.

Very limited information exists on burn injuries and post burn deformities, risk factor associated with knowledge, practice and health seeking behavior of people at country level. It naturally demands an alternative sampling approach. Therefore, the research team decided to develop hospital-based sample plan to overcome the pitfalls in the sample plan inherent in the national surveys for burn injuries. Health Service Providers involved in the management of burn injuries were selected for interview using judgmental sampling technique. The design maintains equal sample size for all hospitals irrespective of their catchment population size since population size is not an important determinant of sample size.^5^

**Summary of tools and techniques used for qualitative data collection**: Researchers approached different groups of people for exploring and validated the content of qualitative information as follows:

**Table:** 

Methods	Participants	Purposes	Number
KII	Medical Superintendents (MS)	Policy and management level gaps in burn injuries related care and services	10
KII	Burn Unit In-charge (Doctors), Nursing Chief and Staff Nurses	Execution of burn injuries and its deformity related care and management through service providers	20
KII	Medical Recorder	Execution of burn injuries related recording and reporting system at hospital level	10
FGD	Community people (Male and Female groups) including FCHVs	Explore the KAP of general population	18 groups (146 participants)

Altogether 40 KIIs (100% response rate) and 18 FGDs (90% response rate) were conducted in the 10 sampled hospitals. FDGs were conducted covering gender, different ethnic groups and occupation at community level. A total of 18 FGDs (9 from Male groups and 9 from Female groups) were undertaken with a total of 146 participants (71 male and 75 female).

For the qualitative method, a content analysis was carried out (transcription and the notes takers' notes were matched). Qualitative data obtained from KIIs and FGDs were analyzed using At. Lasti Software. The findings were presented in narrative forms, comparative charts and quotations. Field work commenced after obtaining ethical approval from Nepal Health Research Council (NHRC).

## RESULTS

Triangulation approach was used to record and analyze the opinions and perceptions of a range of stakeholders about access, availability and barriers to burn injury treatment, care and services. The following themes were extracted from Key Informants Interview (KIIs) with Medical Superintendents, Doctors, Nursing Chief and staff and Medical Recorder and Focus Group Discussions with Male and female groups.

Distribution of burn patients by hospitals: According to the Medical Superintendents (MS), 7 out of ten hospitals have separate burn unit but only 4–5 beds available for burn care management. As reported by the Medical Director of SKMH, in an average, 20–25 cases (90,000–100,000 cases/year) are registered each day in the OPD of which 2–3 cases are burn related. All the KII and FGDs highlighted the fact that majority of the burn patient came from either the adjoining districts or across the country (in case of Bir Hospital, TUTH and SKMH).

The number of burn patients registered in the hospital is inaccurate and under reported. According to Medical Superintendent of Bharatpur Hospital, “Patients having post-burn deformities mostly get treated in the orthopedic department and since there is no separate section to report burn injury in HMIS, the data instead gets reported under deformities and injury.”

**Burn Injuries by type of incidents and treatment received:** From Narayani Sub-Regional Hospital, Birgunj, majorities of male and female FGD participants expressed that an intentional burn injury occurs less in comparison to unintentional. Reasons for intentional burn injury are usually conflicts within family or community, such as couple or friends, whereas unintentional burn might occur due to various reasons.

“Mostly suicidal burn cases of female due to flame/ scald are admitted to the hospital of which 90% cases are referral cases. Cases like fresh burn (acute burn) or patients being primarily treated from other hospitals with Post Burn Contractures (PBC) and scar are handled in this hospital”, said medical staff (doctors, nursing chief and other staff of SKMH)

**Knowledge, practice and treatment seeking behavior of burn patients after injuries:** More than three-fourth (80%) of the hospitals reported that the community people seek home remedy first rather than medical treatment. About one-third male and female from FGD reported that they primarily rely on home remedy and only visit hospital for further treatment if required. Majority of FGD participants reported using oil (sesame), aloe vera, tomatoes, ghee (butter) paste made from medicinal plants, ice/hailstone, honey, potato, toothpaste, moist mud, and water of Hukka (smoking vessel) in case of minor burns. In case of flame burn, cotton cloths and blankets were used to cover the burnt part as a part of primary treatment at home. Usually home remedies are applied to reduce burning sensation, pain and avoid the formation of blisters.

“There is no role of traditional healers amongst the younger generation. Visiting traditional healers was practiced earlier when there was no specific medicine and treatment for burn injuries. However, this practice has been changed in present days”. According to Bharatpur Hospital, Chitwan and Seti Zonal Hospital, Dhangadhi, Kailali.

**Handling critically burn patients before taking to the Hospital:** The doctors, nursing chief and other staff of all ten study hospitals reported that majority of the burn cases are referred to hospital after the first aid treatment (applying silver sulfadiazine cream) at the health posts or druggist/pharmacists. In many instances, burn patients are brought to the hospital late, when wounds have already started to smell. In most of the study sites, community people did not seem to have sufficient knowledge about handling burn injuries (both minor and severe). Very few reported that in case of severe burn, they immediately sent the patients to the hospital in an ambulance/private vehicle or local transportation.

“Burn patients from areas that are hard to reach are referred from community level health facilities to the district or Zonal hospitals after fluid management, TT (Inj. Tetanus toxiod) and antibiotics. Heath workers send those patients with full safety measures in an ambulance to prevent infection, while some who come directly are infected as they come with open wounds”, as reported by medical staff from SKMH.

**Service provision and effectiveness:** Most of the Medical Superintendents mentioned that they provide limited burn related services, but cases are managed either by referring the patients to other hospitals or bringing a team of plastic surgeons at their hospitals. They have limited number of trained staff and higher work load which makes them difficult to manage all type of cases. Also limited physiotherapy services and expensive dressing materials are the barriers in providing quality medical care and treatment for burn patient to prevent further complications and death of burn patients. Most of the referrals are made to the hospitals like TUTH, SKMH or other medical institutions where quality of treatment with trained and experienced staff are available and also offer psychological counseling for both the patients and patient's attendees.

Medical Superintendents of Bheri Zonal Hospital said, “A team of plastic surgeons from Kirtipur hospital visit our hospital periodically to conduct cleft palate surgery and manage severe cases of post burn contractures upon request, whereas contracture cases are referred to Kirtipur hospital directly.”

**Success rate (Chances of survival/death during the treatment):** Majority of the medical doctors, nursing chief, and staff (7 out of 10 hospitals) reported that they mostly manage first degree burn cases that account for around 50% of the total burn cases and 25% belong to second degree with a success rate of 80%. They further added that it is relatively difficult to handle second degree and third degree burn cases than first degree. The success rate is determined by various factors such as age, degree of burn, BSA, nutritional status and other associated diseases like diabetes and hypertension. In addition to that prompt management, routine dressing and willpower of patients also determines the success rate.

As reported by the 7 out of 10 hospitals, the survival rate is 70–75% among total registered burn cases, if burn is more than 40%, success rate reduces to <2%. The hospital could have contributed a higher success rate if they had sufficient resources.

**Experience during burn injury treatment at the Health**

**Facilities/hospitals:** A very less male and female FGD participants expressed that they do not receive quality treatment at the health facilities due to unavailability of trained Service Providers. Though medicines are available, hospital doesn't want to treat the burn patient and immediately refer to other hospitals. Hence, the community people mostly preferred private clinics than government health facilities. FGD participants from area near Bir Hospital and Bheri Zonal Hospital responded that the doctors are always available in the emergency ward and patients receive prompt admission and treatment.

**Complications faced during the treatment:** Medical and Nursing staff reported that hypothermia and sepsis were the major causes of death in most of the burn patients. Risk of failure of multiple organs and burn syndrome was high with burn patients having more than 40% Total Body Surface Area (TBSA). Delayed burn patients having infected wound requires skin grafting.

**Types of deformities developed during treatment:** Deformities like burn contracture, such as hypertrophic scar, keloids, joint stiffness and compartment syndrome are mostly observed during the treatment as reported by the medical staff of the study hospitals. Doctors from Narayani and Gandaki sub-regional Hospital and BPKIHS further reported non-healing chronic ulcers, deformities of joints, fingers, axilla and fist as deformities developed during burn treatment. The findings showed that contractures or scars, either stable or unstable, are developed mostly in patients with second and third-degree burn.

**Challenges faced during treatment and management of the burn injuries in the hospitals:** Almost all the hospital doctors and nurses said that burn injuries are neglected and there should be burn care unit in all major hospitals of Nepal. Human Resource management was another barrier/issue for treatment of burns such as, irregularity of staff in the health facilities/primary health care centers. Also, lack of beds, ICU, ventilators, isolation ward, bathroom and poor Infection Prevention (IP) management makes it difficult for the medical personnel to manage the burn cases. Most of the patients visiting government hospitals belong to poor socio-economic background hence it is likely for them to develop post burn contractures and deformities as they are unable to afford physiotherapy or other post burn procedures. Hence, the Government must implement separate program in order to support the poor and vulnerable.

“For the management of burn injuries, multi sector approach is needed. It's a team work and doctor, nurse, dietician, physiotherapist and psychiatric human resources are also equally important. It is difficult to provide quality care to burn patients with limited human resources”, said Medical Superintendent of Bir Hospital.

**Gender discrimination and patient non-cooperation:** The hospital's staff reported negative or non-cooperative attitudes of the burn patients and their attendants. The nursing In-charge of Narayani SR Hospital informed that female burn victims are brought late to the hospital compared to the male patients. Female patients mostly suffer from physical and mental trauma because of our conservative society. False reporting about the history or incident of the female patient is usually done by her attendants.

**Lack of training to the service providers on burn care management:** Majority of the nurses from the study hospitals reported that they have not received training on counseling and special burn care management. Nurses from TUTH informed that staffs from burn unit receive less training compared to other departments. They further emphasized that training related to burn management must be provided to all staff for better management of burn cases.

**Suggestion for immediate and effective treatment and management of burn injuries and deformities (contracture):** Majority of the doctors, nurses and other staff including medical superintendents highlighted the fact that separate ward for burn patients with sufficient number of beds, physiotherapy ward is required for an effective treatment and management of burn deformities. Also training, capacity building and skill enhancement programs using updated technology for medical personnel will also help to enhance the quality of treatment. They further added that the patients that could not be treated should be referred to the higher center immediately. Moreover, collaboration with development partners would be crucial for training and other support for burn patients.

**Need a policy and practice in place related to burn injuries care and management:** A majority of the hospital authorities as well as service providers reported that there should be a policy and guidelines on burn injury management and post burn contractures and deformities. Government should bring proper law regarding burn injuries (Policy level changes in Health Policy) is important and includes separate recording and reporting system in HMIS, skill and knowledge to health workers and formulate proper treatment strategies and guidelines. There should be standard protocol and training package that should be provided among hospital staffs up to FCHVs level. Health education and awareness program need to be developed by the government and implemented up to the community level. Burn related prevention; treatment and management should be included in the school curriculum and mass awareness created among community people on burn injuries.

## DISCUSSION

In this study, both Health Service Providers and community people's perception towards burn treatment and outcome were collected from ten major referral hospitals across the country. As reports of burn cases are influenced by various factors, 100 percent responses for all variables were difficult to obtain. The findings of this study are very significant hence it could be used as a future reference for disease prevention programs because there are limited burn care facilities in Nepal like Sushma Koirala Memorial Hospital and Kirtipur Hospital.

In this study, though it was not possible to estimate the population-based estimation of burns, the data indicates a slight female preponderance. This was in accordance with some recent studies^6,7^ where females were found to be more affected from burn injuries. A female predominance can be explained as females in the younger age group are mostly engaged in cooking and wear loose-fitting clothes such as saree, dupatta, etc., which inadvertently catch fire easily.^8^

According to Health Service Providers and Community people, majority of the injuries was unintentional which was consistent with the other findings.^9,10^ Main reasons of intentional burn were recorded as a self-inflicted burns/suicidal attempt due to conflicts among couple which was in line with other studies.^11^

Majority of burn patients admitted in the hospitals had Total Burn Surface Area (TBSA) of less than 20% (partial) which was in accordance with Alavi et al. (2012)^12^ and in contrast with few other studies.^7,13,14^

Most of patients went to the hospital for treatment at first followed by private clinic/pharmacy and some of them used home remedies. This finding was almost similar to the findings of the study conducted by Shanmugakrishnan et al. (2008).^15^

In this study, more than two third (67.9%) of the patients developed contractures and more than half (54.6%) scars. There were only 1.2% unstable scars in the retrospective record review whereas one in every four patients (41.7%) developed scars after burn injury in the prospective study. Although, findings of the studies conducted in Bangladesh, Colombia, Egypt and Pakistan shows that 17% of children with burns have a temporary disability and 18% have a permanent disability.^16^

The average length of hospital stay in majority of burn injured patients with all degrees of burn was found less than one month. However, study of socio-demographic Profile of Burn Cases Admitted in Shri Chhatrapati Shivaji Maharaj General Hospital, Solapur, India (2011) reported that 36% patients had hospital stay for less than one day.^17^

Death was recorded very high in third degree than among first and second degree burn patients which was in accordance with another study by Shanmugakrishnan, et al (2008) where none of the cases with <30% TBSA burns died while all those with >55% TBSA burns died.^15^

The predominant cause of death was cardiac arrest, abdominal complication, wound infection and pneumonia which was observed very similar in other studies.^12^

Majority of the burn patients self-reported that the hospitals provided them quality medical services. To reduce the morbidity and mortality due to burn, few preventive measures are recommended such as immediate transport of patients to the hospital for burn injury treatment and awareness raising campaigns at community level through different channels of communication.

## CONCLUSIONS

The number of patients with burn injuries hospitalized every year is in rising trend and most of them require specialize burn care and surgery. But there are very limited burn care facilities outside of Kathmandu valley. Hence, most of the burn patients visit SKMH followed by BPKIHS for care and treatment services. Due to unavailability of immediate care and timely referral to specialized hospital, morbidity and mortality from burn injuries are still increasing.

Intentional burn injuries occur more in female and main reasons of intentional burn are self-inflicted burns/ suicidal attempt. Although, there is good practice at community level for taking hospital immediately (within 6 hours) after burn injuries in majority of cases and getting first aid treatment at the incident places before reaching to the hospital. The length of hospital stays and death of third degree burn patients are more than first and second degree. Cardiac arrest, abdominal complication, wound infection and pneumonia are found major cause of death among burn injured patients.

A majority of the hospital authorities as well as service providers reported that Government should bring the law regarding burn injuries (Policy level changes in Health Policy is important). There should be standard protocol, guidelines and training package up to FCHVs level. Burn related prevention, treatment and management should be included in the school curriculum and mass awareness on burn injuries and its management amongst people of the community.
